# Visceral adiposity index predicts the conversion of metabolically healthy obesity to an unhealthy phenotype

**DOI:** 10.1371/journal.pone.0179635

**Published:** 2017-06-23

**Authors:** Yu Mi Kang, Chang Hee Jung, Yun Kyung Cho, Jung Eun Jang, Jenie Yoonoo Hwang, Eun Hee Kim, Woo Je Lee, Joong-Yeol Park, Hong-Kyu Kim

**Affiliations:** 1 Department of Internal Medicine, Asan Medical Center, University of Ulsan College of Medicine, Seoul, Republic of Korea; 2 Department of Health Screening and Promotion Center, Asan Medical Center, University of Ulsan College of Medicine, Seoul, Republic of Korea; Medical University of Vienna, AUSTRIA

## Abstract

**Objective:**

Some individuals with metabolically healthy obesity (MHO) convert to metabolically unhealthy obesity (MUO) phenotype, and visceral adiposity is one of proposed mechanisms underlying such conversion. Visceral adipose index (VAI) is a novel mathematical model which estimates visceral adiposity based on anthropometric and lipid profiles. We aimed to determine the association of VAI-estimated visceral adiposity with the MHO-to-MUO conversion and the predictive value of VAI in estimating such unfavorable outcomes.

**Methods:**

A total of 2,204 Korean subjects with the MHO phenotype were enrolled and stratified by body mass index and metabolic health state according to Wildman criteria at baseline and last follow-up examinations. VAI was calculated at baseline.

**Results:**

Over a median follow-up period of 41.1 months, 46.0% of subjects converted to MUO phenotype. Higher VAI quartiles were associated with a greater proportion of subjects who underwent MHO-to-MUO conversion, and also with increased odds ratios for such conversion even after multivariate analyses. The optimal VAI cut off value was around 1.00, and VAI had a greater power in the prediction of MHO-to MUO conversion than waist circumference in both genders.

**Conclusion:**

MHO phenotypes with high VAI values are associated with poor future metabolic outcomes. VAI-estimated visceral adiposity is well correlated with the prognosis of MHO subjects, and VAI has a good predictive value in determining the MHO-to-MUO conversion.

## Introduction

Recently, a unique obesity phenotype called the “metabolically healthy obesity (MHO)” state was introduced, which displays favorable cardiometabolic profiles and similar risk of cardiovascular morbidity and mortality compared to normal weight individuals [1–3]. However, the long-term prognosis of MHO state is still in question [4–6]. Although these contradictory findings might be due to differences in ethnicity, age, or inconsistent definitions of the MHO state, the dynamic nature of MHO state might contribute to this controversy [7]. In one study with a median follow-up of 8.2 years, for instance, the MHO individuals were at a higher risk of developing unhealthy metabolic profiles than the metabolically healthy normal-weight individuals[8]. Therefore, for some individuals, the MHO phenotype might be a transient state before its progression to the metabolically unhealthy obesity (MUO) state [8].

A complex interconnection among genetic, environmental, and behavioral factors is thought to be the underlying mechanism of MHO phenotype [9]. Proposed features of the preserved metabolic health in the MHO state include a healthier lifestyle, greater incretin response to meals, less abdominal fat distribution, less visceral and ectopic fat accumulation, lower levels of inflammation, and greater insulin sensitivity [10]. Therefore, sustaining such factors in MHO individuals might prevent the progression to a MUO phenotype.

Among multiple factors that might contribute to metabolic health in the MHO state, one of the most frequently reported key characteristics is the reduced accumulation of visceral fat [11]. A more peripheral fat distribution has been observed in individuals whose MHO phenotype persisted [8], and visceral abdominal fat accumulation detected by a single 10-mm slice computed tomography (CT) scan predicted the conversion of MHO subjects to MUO state after 10 years of follow-up [12]. Therefore, finding a more convenient, well-validated tool for evaluating visceral adiposity might help screen individuals at risk.

Visceral adiposity index (VAI) is a reliable formula, serving as an useful indicator of visceral fat function associated with cardiometabolic risk that does not require costly and inconvenient imaging studies [13]. This simple surrogate marker for visceral adiposity and visceral adipose dysfunction was validated with abdominal magnetic resonance image (MRI) findings and its increase was strongly associated with cardiometabolic risk factors in 1,498 primary care patients [13]. Based on such clinical value of VAI, we hypothesized that VAI-estimated visceral adiposity would be associated with the conversion to MUO state in a large number of subjects who were MHO at baseline. In addition, if such a relationship existed, we aimed to extrapolate the cut-off value of VAI for predicting the MHO-to-MUO conversion.

## Methods

### Study population

Study subjects were recruited from those individuals aged more than 20 years old who visited the Health Screening and Promotion Center of Asan Medical Center (Seoul, Republic of Korea) between March 2007 and December 2013 for routine medical examinations. During this period, 46,490 subjects received medical examinations on at least two occasions. Among the total of 46,490 subjects, we excluded 2,791 subjects who had taken drugs that could potentially affect lipid metabolism for more than 6 months or within the previous 12 months at a baseline examination. We also excluded 987 subjects with a history of cardiovascular disease (CVD) at baseline examination. In addition, subjects with the absence of data (insulin level; n = 4.759, and hsCRP level; n = 272) were excluded. Finally, subjects with a high-sensitivity C-reactive protein (hsCRP) level greater than 10 mg/L (n = 623) were omitted to exclude occult infection or other systemic inflammatory processes [14]. Several subjects met more than two criteria. After the exclusion of ineligible subjects, 2,204 subjects (1,576 men and 628 women) with a mean age of 47.1 years (range 20–76 years) who showed MHO phenotype at baseline out of 38,166 were enrolled. Subjects were followed for a median of 41.1 months with a range of 6.0–80.5 months. All subjects provided written informed consent. This study was approved by the Institutional Review Board of Asan Medical Center.

All subjects completed a questionnaire on their previous medical and/or surgical diseases, medications, and drinking and smoking habits. Drinking habits were categorized as frequency per week (i.e., ≤ once/week and ≥ twice/week [moderate drinker]); smoking habits as noncurrent or current; and exercise habits as frequency per week (i.e., ≤ twice/week and ≥3 times/week [physically active]) [15]. Postmenopausal status was defined as the cessation of menses for ≥1 year, confirmed by a serum follicle-stimulating hormone concentration of >30 IU/L.

### Clinical and laboratory measurements

Anthropometric examinations were performed while subjects were wearing light-weight clothing provided by the institution with shoes removed. The body mass index (BMI) was calculated by dividing the weight in kilograms by the square of the height in meters. The waist circumference (WC, cm) was measured midway between the costal margin and the iliac crest at the end of a normal expiration. Following a resting period of at least 15 minutes, blood pressure (BP) was measured on the right arm by automatic manometry using a Vital Sign Monitor 300 Series (Welch Allyn Co., Ltd., Beaverton, OR) with an appropriate cuff size. The measured BP was the average value of the three consecutive readings, with a 5-minute break between each measurement. After overnight fasting, blood samples were drawn from subjects’ antecubital veins into vacuum-sealed tubes and were transferred to a central, certified laboratory at Asan Medical Center. Measurements included the concentration of fasting glucose, insulin, hsCRP, several lipid parameters, and liver enzymes.

Fasting total cholesterol, high-density lipoprotein-cholesterol (HDL-C), low-density lipoprotein-cholesterol (LDL-C), triglycerides (TG), uric acid, aspartate aminotransferase (AST), and alanine aminotransferase (ALT) were measured by an enzymatic colorimetric method using a Toshiba 200 FR Neo autoanalyzer (Toshiba Medical System Co., Ltd., Tokyo, Japan). Gamma-glutamyltransferase (GGT) was measured using the L-γ-glutamyl-p-nitroanilide method (Toshiba). HsCRP and fasting plasma glucose (FPG) were measured using the immunoturbidimetric method (Toshiba) and by an enzymatic colorimetric method using a Toshiba 200 FR autoanalyzer (Toshiba), respectively. Serum insulin was measured by immunoradiometric assay (TFB Co., Ltd., Tokyo, Japan). Ion-exchange high-performance liquid chromatography (Bio-Rad Laboratories, Inc., Hercules, CA) was used to measure HbA1c levels. The intra- and inter-assay coefficients of variation of these analyses were consistently <3.5%. The homeostatic model assessment of insulin resistance (HOMA-IR) was calculated as the product of the fasting serum insulin (μU/mL) and FPG (mmol) concentrations divided by 22.5. All enzyme activities were measured at 37°C.

### Definitions of metabolic health and obesity states

In this study, Asia-Pacific BMI criteria (non-obesity<25 kg/m^2^ and obesity ≥25 kg/m^2^) that were established by the World Health Organization Western Pacific Region [16] and officially adopted by the Korean Centers for Disease Control and Prevention and other Korean governmental organizations [17] were used to define obesity phenotypes. On the other hand, the consensus on the best standardized definition of obesity phenotypes is still lacking. For the present analyses, metabolically healthy individuals were defined according to Wildman criteria as having none of the following risk factors[18]: (1) a systolic BP ≥ 130 mmHg and/or a diastolic BP ≥ 85 mmHg, or on antihypertensive treatment; (2) TG ≥1.7 mmol/L; (3) FPG ≥5.6 mmol/L (impaired fasting glucose, IFG) and/or taking antidiabetic medications; (4) HDL-C < 1.0 mmol/L in men and <1.3 mmol/L in women; 5) HOMA-IR ≥ 90th percentile (≥ 3.16); and 6) hsCRP ≥ 90th percentile (≥2.1 mg/L). According to these criteria, study participants were categorized into one of four groups: (1) Metabolically healthy, non-obesity (MHNO), BMI <25 kg/m2 and no metabolic risk factors; (2) metabolically unhealthy, non-obesity (MUNO), BMI <25 kg/m2 and ≥1 metabolic risk factors; (3) MHO, BMI ≥25 kg/m2 and no metabolic risk factors; or (4) MUO, BMI ≥25 kg/m2 and ≥1 metabolic risk factors.

### Assessment of visceral adiposity by calculation of VAI

Visceral adiposity was assessed by calculating VAI according to previously validated equations [13]. VAI was defined as the following equations:

$$\mathrm{Men}:\;\mathrm{VAI}=\left(\frac{\mathrm{WC}}{39.68+(1.88\times \mathrm{BMI})}\right)\times \left(\frac{\mathrm{TG}}{1.03}\right)\times \left(\frac{1.31}{\mathrm{HDL-C}}\right)$$

$$\mathrm{Women}:\;\mathrm{VAI}=\left(\frac{\mathrm{WC}}{36.58+(1.89\times \mathrm{BMI})}\right)\times \left(\frac{\mathrm{TG}}{0.81}\right)\times \left(\frac{1.52}{\mathrm{HDL-C}}\right)$$

VAI was formulated assuming a VAI of 1 for healthy subjects without obesity with normal adipose distribution and normal TG and HDL-C levels.

### Statistical analysis

Subjects were categorized into four gender-specific quartile groups according to the baseline VAI values. Continuous variables with normal and skewed distribution are expressed as mean ± SD and median (and interquartile range), respectively. Categorical variables are expressed as proportions (%). The demographic and biochemical characteristics of the study population according to the metabolic health and obesity state at baseline were compared using one-way analysis of variance (ANOVA) with Scheffe’s method as the post-hoc analysis or the Kruskal-Wallis test with the Dunn procedure for continuous variables and the chi-square test for categorical variables as the post-hoc analysis. Student’s *t*-test or the Mann-Whitney U test (for continuous variables) and the chi-squared test (for categorical variables) were used to compare the demographic and biochemical characteristics of the two groups of study subjects (i.e., MHO vs. MUO) at the follow-up examination. The odds ratios (ORs) of conversion to the MUO state in subjects who were originally categorized as MHO at baseline were calculated using multivariate logistic regression analysis, in which various adjustment models consisting of different confounding variables were applied. To assess the utility of VAI as a marker for predicting the MHO-to-MUO conversion, receiver operating characteristics (ROC) curves were constructed and the areas under the curve (AUC) were calculated accordingly. The distance on the ROC curve of VAI was calculated by plotting the sensitivity against (1-specificity). To compare the predictive value of VAI and WC, ROC curve was also constructed for WC and the difference between the AUC was additionally analyzed. The AUC and the crucial points were determined using Med-Calc® version 11.6.1.0 for Windows (MedCalc Software, Mariakerke, Belgium). All other statistical analyses were performed using SPSS version 20.0 for Windows (SPSS Inc., Chicago, IL, USA). A *P*-value < 0.05 was considered statistically significant.

## Results

### Baseline characteristics of the study subjects

The baseline clinical and biochemical characteristics of the total study subjects according to baseline metabolic health and obesity are shown in Table 1. The MHO phenotype represented 5.8% (n = 2,204) of the total participants and 18.5% of the obese population. Compared with subjects categorized as MHNO, MHO individuals were more likely to be male, older, current smoker, moderate drinker, and to have a less favorable risk profile (Table 1). There was no difference in the proportions of ‘physically active’ individuals between the MHO and MHNO groups.

**Table 1:** Baseline clinical and biochemical characteristics of the study subjects according to baseline metabolic health and obesity.

	Non-obese		Obese		
	MHNO	MUNO	MHO	MUO	
Variables	(n = 11,503)	(n = 14,731)	(n = 2,204)	(n = 9,728)	P for trend
**Age (year)**	45.4±8.4	49.6±8.7	47.1±8.3	49.1±8.7	<0.001
**Sex (male, %)**	36.6	61.8	71.5	81.4	<0.001
**BMI (kg/m**^**2**^**)**	21.5±2.1	22.6±1.9	26.5±1.4	27.2±2.0	<0.001
**WC (cm)**	74.7±9.5	79.8±7.0	87.6±9.0	90.7±9.2	<0.001
**Systolic BP (mmHg)**	108.4±10.0	119.4±14.8	114.0±8.3	123.8±13.6	<0.001
**Diastolic BP (mmHg)**	67.6±7.7	75.3±10.6	71.2±6.6	78.6±10.1	<0.001
**Antihypertensive medication (%)**	0.0^a^	14.7	0.0^a^	21.7	<0.001
**Current smoker (%)**	13.6	21.8^a^	22.1^a^	28.1	<0.001
**Moderate drinker (%)**	25.8	39.7	46.0	52.6	<0.001
**Physically active (%)**	37.4^a^	38.8^a^	39.7^a^	35.5	0.002
**Family history of diabetes (%)**	17.8^a^	21.4^b^	17.2^a^	22.1^b^	<0.001
**FPG (mmol/L)**	5.0±0.4	5.6±1.1	5.1±0.3	5.8±1.1	<0.001
**HbA1c (%)**	5.2 (5.0–5.5)	5.4 (5.2–5.7)	5.3 (5.1–5.5)	5.5 (5.3–5.9)	<0.001
**Total cholesterol (mmol/L)**	4.8±0.8	5.0±0.9^a^	5.0±0.8^a^	5.1±0.9	<0.001
**TG (mmol/L)**	0.9 (0.7–1.1)	1.3 (0.9–1.8)	1.1 (0.9–1.3)	1.7 (1.2–2.3)	<0.001
**LDL-C (mmol/L)**	2.9±0.7	3.2±0.8	3.3±0.7	3.3±0.8	<0.001
**HDL-C (mmol/L)**	1.7±0.3	1.4±0.4	1.5±0.3	1.3±0.3	<0.001
**Uric acid (μmol/L)**	4.6±1.2	5.2±1.4	5.5±1.3	6.0±1.4	<0.001
**AST (U/L)**	21 (18–25)	22 (19–27)	23 (19–28)	21 (25–32)	<0.001
**ALT (U/L)**	16 (12–21)	20 (15–27)	22 (16–29)	27 (20–38)	<0.001
**GGT (U/L)**	13 (10–19)	19 (13–31)^a^	20 (14–30)^a^	30 (19–48)	<0.001
**hsCRP (mg/L)**	0.4 (0.3–0.6)	0.6 (0.3–1.2)^a^	0.6 (0.4–0.9)^a^	0.9 (0.5–1.6)	<0.001
**HOMA-IR**	1.04 (0.73–1.44)	1.51 (1.06–2.16)	1.42 (1.03–1.93)	2.22 (1.54–3.20)	<0.001

Data are n (%), median (interquartile range), or mean±SD.

^a,b^Same letters indicate a statistically insignificant difference.

Compared with subjects categorized as MUO, MHO subjects were characterized by younger age, higher degree of physical activity, and lower current rates of smoking and drinking (Table 1). They also showed a more favorable risk profile than MUO (Table 1). Compared with MUNO individuals, these subjects showed a less insulin-resistant profile such as higher HDL-C, lower TG, FPG, HbA1c, and HOMA-IR levels despite their higher BMI and WC (Table 1).

Among the 2,204 MHO subjects in the study, 46.0% (n = 1,104) of subjects converted to the MUO state at their last follow-up visit, whereas 33.6% (n = 740) of subjects remained within the MHO category. A total of 20.4% out of 2,204 MHO subjects converted to non-obese group (12.1% to MHNO and 8.3% to MUNO, respectively). Table 2 shows the baseline clinical and biochemical characteristics of the obese subjects according to the conversion of metabolic health during the study period. Compared with subjects who remained in the MHO category, those who underwent future conversion to MUO were more likely to be male, current smokers, moderate drinkers, and to have a less favorable risk profile at baseline (Table 2). There were no statistically significant differences in the age, the proportions of ‘physically active’ individuals and the presence of family history of diabetes between those who remained in the MHO category and those who underwent conversion to MUO groups at the last follow-up period (Table 2).

**Table 2:** Baseline clinical and biochemical characteristics of the obese subjects according to the conversion of metabolic health during the study period.

	MHO at follow-up	MUO at follow-up	
Variables	(n = 740)	(n = 1,014)	*P* value
**Age (year)**	46.9±8.2	47.1±8.5	0.714
**Sex (male, %)**	67.3	77.1	<0.001
**BMI (kg/m**^**2**^**)**	26.6±1.3	26.9±1.6	<0.001
**WC (cm)**	87.1±10.2	88.9±8.9	<0.001
**Systolic BP (mmHg)**	112.7±8.2	115.5±8.1	<0.001
**Diastolic BP (mmHg)**	70.5±6.6	72.0±6.5	<0.001
**Current smoker (%)**	19.6	25.2	0.006
**Moderate drinker (%)**	40.4	50.6	<0.001
**Physically active (%)**	40.7	38.7	0.401
**Family history of diabetes (%)**	16.2	17.3	0.605
**FPG (mmol/L)**	5.0±0.3	5.1±0.3	<0.001
**HbA1c (%)**	5.3 (5.0–5.5)	5.3 (5.1–5.5)	0.002
**Total cholesterol (mmol/L)**	5.0±0.8	5.1±0.8	0.017
**TG (mmol/L)**	1.0 (0.8–1.3)	1.1 (0.9–1.4)	<0.001
**LDL-C (mmol/L)**	3.2±0.7	3.3±0.7	<0.001
**HDL-C (mmol/L)**	1.5±0.3	1.4±0.3	<0.001
**Uric acid (μmol/L)**	5.3±1.3	5.7±1.3	<0.001
**AST (U/L)**	22 (19–27)	23 (19–28)	0.001
**ALT (U/L)**	21 (15–27)	23 (18–32)	<0.001
**GGT (U/L)**	18 (12–27)	22 (15–33)	<0.001
**hsCRP (mg/L)**	0.5 (0.3–0.9)	0.7 (0.4–1.0)	<0.001
**HOMA-IR**	1.36 (0.98–1.84)	1.48 (1.09–2.01)	<0.001

Data are n (%), median (interquartile range), or mean±SD.

### Incidence of MHO-to-MUO conversion according to VAI quartiles

**Fig 1:**
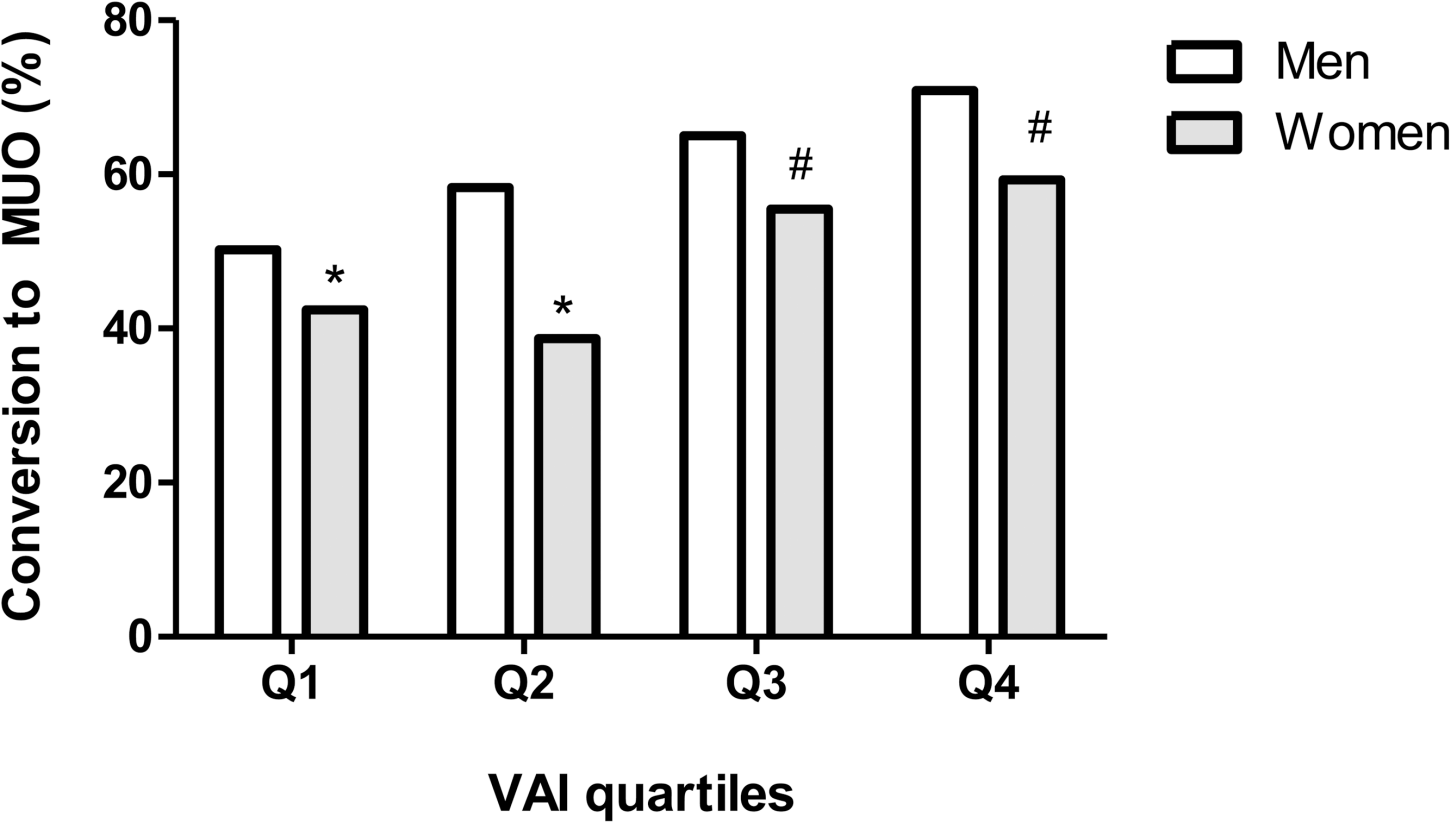
Crude incidence of MUO conversion from MHO state according to VAI quartiles. Same symbols indicate a statistically insignificant difference.

During the median follow-up period of 41.1 months, the crude incidence rates of MUO conversion according to VAI quartiles in men were 50.2% in Q1, 58.3% in Q2, 65.0% in Q3, and 70.9% in Q4, showing that an increase in the VAI quartiles was associated with an increased incidence of MUO conversion from MHO state (Fig 1). In women, the crude incidence rates of MUO conversion according to VAI quartiles were 42.4% in Q1, 38.7% in Q2, 55.5% in Q3, and 59.3% in Q4 (Fig 1).

Table 3 shows the ORs for progression to MUO from MHO state in men. Even after adjusting for multiple confounding variables, the ORs for progression to MUO from MHO state was significantly higher in subjects exhibiting higher VAI quartiles. When compared to the first quartile (Q1) of VAI, the ORs of MUO progression in the second (Q2), third (Q3), and fourth quartiles (Q4) were 1.36 (95% CI 0.99–1.87), 1.79 (95% CI 1.29−2.49), and 2.26 (95% CI 1.60−3.17), respectively (Model 2 in Table 3). In women, similar significant pattern was observed, although the degree of ORs of higher VAI quartiles was less pronounced compared with men (Model 2 in Table 4). Other variables which were associated with progression to MUO from MHO state were uric acid, and follow-up months (Table 3 and Table 4). Additionally, menopause state was one of significant contributors to the conversion MHO-to-MUO state in women (Table 4).

**Table 3:** Odds ratios (ORs) and 95% confidence intervals (CI) for progression to MUO based on VAI quartile categories in men.

Variables	Unadjusted	Model 1	Model 2
VAI			
Q1 (≤0.7961)	1.00 (Reference)	1.00 (Reference)	1.00 (Reference)
Q2 (0.7962–1.0733)	1.39 (1.02–1.90)	1.38 (1.01–1.89)	1.36 (0.99–1.87)
Q3 (1.0734–1.3545)	1.85 (1.34–2.54)	1.83 (1.33–2.52)	1.79 (1.29–2.49)
Q4 (≥1.3546)	2.43 (1.75–3.36)	2.43 (1.74–3.38)	2.26 (1.60–3.17)
Age		1.00 (0.99–1.02)	1.01 (0.99–1.02)
Moderate drinker		1.42 (1.13–1.79)	1.30 (0.99–1.67)
Current smoker		1.04 (0.80–1.35)	1.06 (0.81–1.39)
Physically active		0.94 (0.74–1.19)	0.89 (0.69–1.13)
uric acid			1.15 (1.03–1.28)
AST			1.01 (0.98–1.03)
ALT			1.01 (0.99–1.02)
GGT			1.00 (0.98–1.01)
follow-up months			1.02 (1.01–1.02)

**Table 4:** Odds ratios (ORs) and 95% confidence intervals (CI) for progression to MUO based on VAI quartile categories in women.

Variables	Unadjusted	Model 1	Model 2
VAI			
Q1 (≤0.8132)	1.00 (Reference)	1.00 (Reference)	1.00 (Reference)
Q2 (0.8133–1.0545)	0.86 (0.51–1.44)	0.84 (0.50–1.42)	0.82 (0.48–1.40)
Q3 (1.0546–1.4306)	1.69 (1.01–2.83)	1.66 (0.99–2.78)	1.49 (0.87–2.57)
Q4 (≥1.4307)	1.98 (1.18–3.33)	1.87 (1.11–3.16)	1.75 (1.02–3.01)
Age		1.02 (0.99–1.05)	1.05 (0.98–1.09)
Moderate drinker		1.09 (0.63–1.88)	0.95 (0.54–1.69)
Current smoker		1.53 (0.44–5.33)	1.26 (0.35–4.57)
Physically active		0.90 (0.62–1.32)	0.87 (0.58–1.29)
Menopause state		1.23 (1.13–1.26)	1.12 (1.09–1.24)
uric acid			1.36 (1.09–1.68)
AST			1.00 (0.96–1.04)
ALT			1.00 (0.98–1.02)
GGT			1.03 (0.99–1.05)
follow-up months			1.02 (1.01–1.03)

### Predictive value of VAI in determining the future conversion to MUO

**Fig 2:**
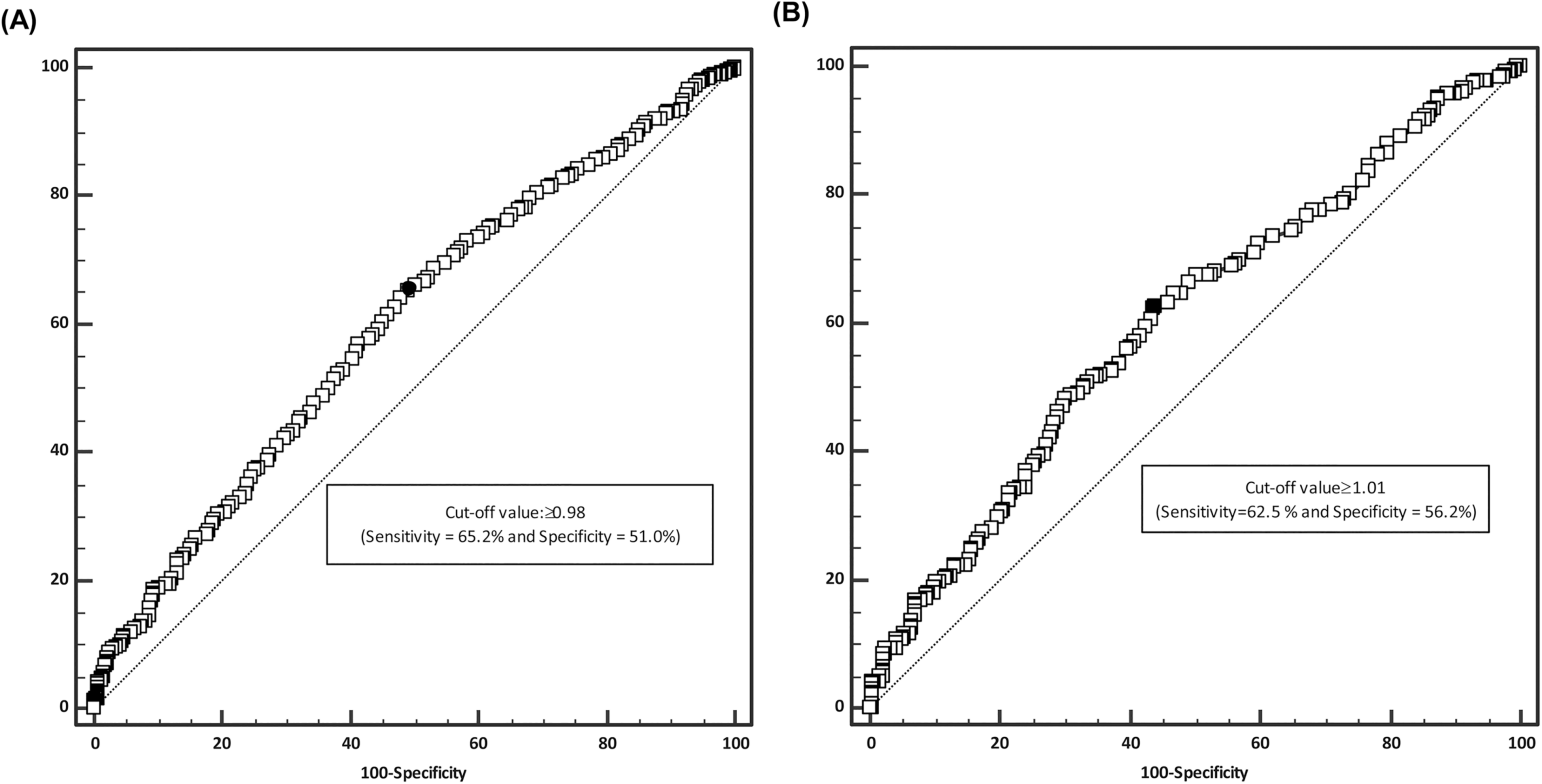
**Receiver operating characteristic (ROC) curve and optimal VAI cutoff value for determining the future conversion to MUO from MHO state in men (A) and in women (B)**.

ROC analysis revealed that the optimal VAI cutoff for determining the future conversion to MUO was 0.98, which had a sensitivity of 65.2% and a specificity of 51.0% (Fig 2A, AUC, 0.600; 95% CI, 0.566–0.629, *P* < 0.001) in men, while 1.01, which had a sensitivity of 62.5% and a specificity of 56.2% (Fig 2B, AUC, 0.603; 95% CI, 0.552–0.654, *P*<0.001) in women. Using this cut-off value, the ORs for future conversion to MUO from baseline MHO state were 1.88 (95% CI, 1.48–2.38, *P* < 0.001) in men after adjusting for age, smoking and drinking habits, physical activities, uric acid, AST, ALT, GGT, and follow-up months and 1.91 (95% CI, 1.30–2.82, *P* < 0.001) in women after further adjusting for menopause state (Data not shown). VAI had a greater predictive power than that of WC (AUC, 0.558; 95% CI, 0.526–0.590, *P* < 0.001; Table 5) when additional ROC analyses were performed for comparison (differences between areas, 0.039; 95% CI, 0.002–0.080, *P* = 0.038; Table 5) in men. The similar pattern was observed in women (differences between areas, 0.057; 95% CI, 0.011–0.124, *P* = 0.042; Table 5).

**Table 5:** Prediction of MHO-to-MUO conversion with VAI and WC.

Predictor of visceral adiposity	AUC	95% CI	*P*-value	Difference between AUC	95% CI	*P*-value^a^
**Men**						
VAI	0.600	0.566–0.629	<0.001	0.039	0.002–0.080	0.038
WC	0.558	0.526–0.590	<0.001			
**Women**						
VAI	0.603	0.552–0.654	<0.001	0.057	0.011–0.124	0.042
WC	0.546	0.495–0.598	0.080			

^a^*P*-value for comparison between VAI and WC.

## Discussion

A substantial amount of evidence refuting the benign nature of the MHO phenotype has been recently reported in literature and its long-term prognosis has been debated [19–21]. To explain such an unfavorable prognosis, it was suggested that, for some individuals, the MHO phenotype might be a transient state before their progression to MUO state [8]. In line with this suggestion, approximately a half of the MHO subjects converted to the MUO phenotype after the median follow-up of 41.1 months, whereas 33.6% remained in the MHO state at follow-up in our current study. VAI was well correlated with the incidence of conversion to the MUO phenotype, and the ORs for such metabolic deterioration significantly increased with higher VAI quartiles, even after multivariate analysis (Table 3 and Table 4). Our study results suggest that the MHO phenotype is a transient state in some individuals and that visceral adiposity and visceral fat distribution estimated by VAI predict the future metabolic deterioration of subjects who initially displayed MHO phenotype.

Of multiple factors that might contribute to metabolic health [10], the most frequently reported key characteristics of the MHO phenotype are reduced accumulation of visceral and ectopic fat and higher insulin sensitivity [22–24]. Similarly, a greater peripheral fat distribution has been observed in individuals with a persistent MHO phenotype [8], and visceral abdominal fat accumulation detected by a single 10-mm slice CT scan predicted the conversion of MHO subjects to MUO state after 10 years of follow-up [12]. Although the influence of visceral fat accumulation on one’s metabolic health is complex, it is primarily thought that the expandability of subcutaneous fat is important in maintaining the MHO phenotype. It has been postulated that the storage capacity of adipocytes might be exceeded, leading to ectopic accumulation of lipids (i.e. in visceral fat depots, liver, muscle and β-cells) in MUO individuals, in contrast to MHO individuals whose subcutaneous adipose tissue might have greater intrinsic propensity to expand, leading to relatively higher insulin sensitivity [10]. Based on these findings, therefore, attempts were made to identify a useful tool to evaluate visceral adiposity to help screen individuals at risk and enable a timely intervention. However, to date, fat distribution and visceral adiposity have been measured by imaging studies such as CT, MRI scans, and dual-energy X-ray absorptiometry (DXA) [25, 26].

The degree of the contribution of visceral adiposity to metabolic deterioration is well known from the results of previous cross-sectional studies [27–29]. For instance, Peppa et al. suggested that DXA-derived centrality ratios such as trunk-to-legs and abdominal-to-gluteofemoral fat ratio could effectively discriminate between subtypes of obesity in obese postmenopausal women [30]. However, one prospective longitudinal study of Japanese-American adults with obesity (i.e. BMI≥25kg/m^2^) recently reported that approximately two-thirds of the MHO population underwent conversion to the MUO phenotype after 10 years of follow-up [12]. In that study, a univariate logistic regression analysis revealed that fasting plasma insulin, HOMA-IR and TG levels as well as subcutaneous abdominal fat (SAT) and visceral abdominal fat (VAT) directly measured by CT were positively associated with the development of MUO phenotype [12]. Although the protective effect of SAT in MHO individuals was not demonstrated, possibly due to the longitudinal nature and a relatively small number of MHO subjects in that study, VAT was well associated with the future metabolic deterioration (OR per 1-s.d. increment [95% CI], 2.04 [1.11–3.72], *P* = 0.021) [12]. Similarly, in our current study, almost a half of the MHO subjects underwent metabolic deterioration within a shorter time period (i.e. median follow-up of 41.1 months), and the VAI-estimated visceral fat distribution was a useful determinant of such phenotypic alteration (Tables 3 and 4 and Fig 2). Notably, both studies observed the deteriorative effect of visceral adiposity in a longitudinal manner; however, VAI exempts one from the necessity of taking high-cost imaging studies, thereby making the prediction much more practical in a clinical setting.

VAI is a gender-specific mathematical model, which originates from the observation in a healthy normal/overweight population of a linear relationship between BMI and WC, from which a linear equation has been extrapolated [31]. VAI was further validated with abdominal MRI findings and it exhibited a strong association with both the rate of peripheral glucose utilization during the euglycemic–hyperinsulinemic clamp [13]. Theoretically, therefore, VAI should be more effective in predicting the metabolic outcome in MHO individuals. Likewise, we found that VAI has a greater predictive power than that of WC by performing additional ROC analyses for comparison (Table 5). Thus, VAI can be a more appropriate candidate as a surrogate measure for predicting unfavorable metabolic outcomes in MHO individuals than WC, possibly due to a more accurate assessment of visceral adiposity.

Upper body fat accumulation, decreased peripheral fat deposition and ectopic fat storage are the examples of altered body composition during the transition to menopause, and these alterations are considered as the major mediator of menopause-related cardiometabolic morbidity and mortality [32, 33]. In accordance with this, menopause status was one of the contributing factors in the conversion of MHO to MUO state in women of our current study (Table 4). The findings that the degree of ORs of MHO-to-MUO conversion in women of higher VAI quartiles was less pronounced compared with men (Table 3 and Table 4) might be due to this substantial effect of menopause on the body fat distribution. However, the results in which VAI showed the independent association with MHO-to-MUO conversion further supported the importance of visceral adiposity, regardless of the menopause status (Table 4).

In our analysis, uric acid was one of the independent factors associated with the conversion of MHO to MUO state in both genders (OR 1.15, 95% CI 1.03–1.28 in men and OR 1.36, 95% CI 1.09–1.68 in women; Table 3 and Table 4). In a previous study designed to distinguish metabolically healthy from unhealthy overweight/obese young and adult patients, serum levels of uric acid were suggested as a considerable discriminator between those two obesity phenotypes [34]. Although it is still controversial whether uric acid is beneficial per se or just and innocent bystander in various disease conditions [34], the results of our study further support the role of uric acid as a possible biomarker to distinguish obese phenotypes.

Because the mathematical modeling process of VAI was performed based on the data of healthy Caucasian men and women, the usefulness of VAI in an Asian population such as the subjects of our current study has been unclear [13, 31]. However, a previous study with young Korean women with polycystic ovary syndrome has shown a positive correlation of VAI with the visceral fat area and the visceral distribution of adiposity measured with CT [35]. Likewise, our present study results showed a strong correlation between higher VAI quartiles and future metabolic derangement (Tables 3 and 4 and Fig 1), and its appropriate application in our population was further suggested by the ROC curve analysis, which indicated a cut-off value of around 1.00 might effectively predict the metabolic deterioration of MHO individuals in the future (Fig 2). Taken together, we speculate that VAI can serve as a useful surrogate marker to predict the long-term cardiometabolic outcomes in individuals with the MHO phenotype. To our knowledge, our current study is one of the largest studies to date to evaluate the association of visceral adiposity with the future conversion of MHO individuals to the MUO phenotype, and it is the first to prove that VAI alone without the need for imaging studies can serve as a reliable marker to predict this conversion.

There are several limitations in this study. First, despite the large cohort size, our subjects were not necessarily representative of the general Korean or Asian population due to the voluntary nature of the recruitment. Second, the follow-up period in our current study was broad (6.0–80.5 months). This might underestimate or overestimate the conversion rate of the obese phenotypes, although we adjusted the follow-up months in our multivariate logistic regression analysis (Table 3 and Table 4). Third, imaging studies for fat distribution was not available in most patients, and thus, the direct comparison between VAI and physical quantification of fat distribution could not be made. However, VAI was previously well-validated by MRI findings and its retrospective association with cardiometabolic outcomes [13, 36]. Fourth, although many baseline metabolic markers are significantly different between MHO and MUO at follow-up (Table 2), these differences were small and may be most likely attributable to the large sample size which increased statistical power. Lastly, we did not evaluate hard outcomes such as mortality and major cardiovascular events associated with the conversion from MHO to the MUO phenotype.

## Conclusions

In conclusion, a substantial proportion of MHO individuals cannot maintain healthy cardiometabolic outcomes and subsequently convert to the MUO phenotype in the near future. The visceral adiposity evaluated by VAI correlates well with this metabolic deterioration. The availability of visceral adiposity evaluation without costly and time-consuming imaging studies might be more practical in a clinical setting. However, future prospective longitudinal studies investigating the predictive value of VAI in determining the composite metabolic outcomes of MHO individuals with a longer-term follow-up period are warranted.

## Abbreviations

ALT: Alanine aminotransferase
AST: aspartate aminotransferase
CVD: cardiovascular disease
DBP: diastolic blood pressure
FPG: fasting plasma glucose
GGT: Gamma-glutamyltransferase
HDL-C: HDL-cholesterol
HOMA-IR: homeostatic model assessment of insulin resistance
hsCRP: high-sensitivity C-reactive protein
LDL-C: LDL-cholesterol
MHNO: metabolically healthy non-obesity
MHO: metabolically healthy obesity
MUNO: metabolically unhealthy non-obesity
MUO: metabolically unhealthy obesity
SBP: systolic blood pressure
TG: triglycerides
VAI: visceral adiposity index
WC: waist circumference
